# Seeking the environmental source of Leptospirosis reveals durable bacterial viability in river soils

**DOI:** 10.1371/journal.pntd.0005414

**Published:** 2017-02-27

**Authors:** Roman Thibeaux, Sophie Geroult, Claire Benezech, Stéphane Chabaud, Marie-Estelle Soupé-Gilbert, Dominique Girault, Emilie Bierque, Cyrille Goarant

**Affiliations:** 1 Institut Pasteur in New Caledonia, Institut Pasteur International Network, Leptospirosis Research and Expertise Unit, Noumea, New Caledonia; 2 Health Department, Direction of Health and Social Affairs of New Caledonia, Noumea, New Caledonia; University of Minnesota, UNITED STATES

## Abstract

**Background:**

Leptospirosis is an important re-emerging infectious disease that affects humans worldwide. Infection occurs from indirect environment-mediated exposure to pathogenic leptospires through contaminated watered environments. The ability of pathogenic leptospires to persist in the aqueous environment is a key factor in transmission to new hosts. Hence, an effort was made to detect pathogenic leptospires in complex environmental samples, to genotype positive samples and to assess leptospiral viability over time.

**Methodology/Principal findings:**

We focused our study on human leptospirosis cases infected with the New Caledonian *Leptospira interrogans* serovar Pyrogenes. Epidemiologically related to freshwater contaminations, this strain is responsible for ca. 25% of human cases in New Caledonia. We screened soil and water samples retrieved from suspected environmental infection sites for the pathogen-specific leptospiral gene *lipL-32*. Soil samples from all suspected infection sites tested showed detectable levels of pathogenic leptospiral DNA. More importantly, we demonstrated by viability qPCR that those pathogenic leptospires were viable and persisted in infection sites for several weeks after the index contamination event. Further, molecular phylogenetic analyses of the leptospiral *lfb-1* gene successfully linked the identity of environmental *Leptospira* to the corresponding human-infecting strain.

**Conclusions/Significance:**

Altogether, this study illustrates the potential of quantitative viability-PCR assay for the rapid detection of viable leptospires in environmental samples, which might open avenues to strategies aimed at assessing environmental risk.

## Author summary

Leptospirosis is an emerging zoonotic disease caused by infection with pathogenic strains of *Leptospira*. Most human infections arise from environmental exposure to contaminated freshwater environments or watered soils where pathogenic *Leptospira* are considered as able to survive for prolonged periods. Therefore, a good understanding of *Leptospira* survival strategy in the environment is a key step to identifying crucial factors amenable to interventions and public health actions to lower leptospirosis burden. In this study, we investigated the environmental presence and survival of pathogenic leptospires in areas where recent human leptospirosis cases had been reported. Although detection of *Leptospira* from complex environmental samples is difficult, we successfully detected the presence of pathogenic *Leptospira* in soils of suspected infection sites. In addition, we showed that these pathogenic leptospires were alive and present in soils several weeks after the infecting event. Typing of leptospiral DNA retrieved from the environment revealed identities between environmental pathogenic *Leptospira* and the causative strains involved in human leptospirosis index cases. Interestingly, we also identified yet unreported genotypes. Altogether, our work illustrates the potential of quantitative molecular assays for the rapid detection and typing of viable leptospires in environmental samples, which could prove useful to assess the risk of environmental exposure.

## Introduction

Leptospirosis is an acute febrile disease caused by pathogenic spirochetes of the genus *Leptospira*. It is considered an important re-emerging infectious disease that affects more than 1 million humans worldwide [1]. The spectrum of human disease caused by leptospires is extremely wide, ranging from subclinical infection to a severe syndrome of multiorgan infection with high mortality. *Leptospira* transmission from the urine of reservoir hosts to incidental hosts, including humans, usually occurs through the contamination of skin lesions or mucosae with contaminated surface water or soil [2]. The incidence of such infections depends on several factors including the density of the reservoir species and its *Leptospira* carriage prevalence, the dilution into watered environment and the survival time of the leptospires into possibly nutrient-poor and adverse environmental conditions. Estimation of survival time and virulence preservation of pathogenic *Leptospira spp*. after excretion into the environment is becoming a crucial challenge to determine the environmental risk and to adopt preventive measures. The duration of *Leptospira* survival in natural habitat is affected by many factors including abiotic and biotic factors. The persistence of pathogenic *Leptospira* in moist soil and freshwater for long periods of time is thought to depend on a slightly alkaline pH, high oxygen, and low salt concentrations [3–5]. The classical assumption is that slightly higher alkalinity (up to pH 8.0) allows for longer survival. Under laboratory conditions, a strain of serovar Javanica was reported to survive in distilled water (pH 7.8) for 152 days [6]. More recently, Andre-Fontaine *et al*. [7] showed that pathogenic *Leptospira* can survive for months in mineral water. Interestingly, *Leptospira* were reported to survive as long as 10 months in adverse conditions (4°C) and up to 20 months when stored at 30°C.

Interactions of *Leptospira spp*. with the environmental microbiota also begin to be examined. Environmental microbial blooms alter the concentration of oxygen, minerals, and other nutrients in the water and favor either multiplication or destruction of some species of pathogenic *Leptospira* [8]. Several common bacterial genera including *Azospirillum* and *Sphingomonas* were found along with pathogenic and saprophytic *Leptospira spp*. in biofilms formed in freshwater or in dental water unit systems [9,10]. Co-incubation with a *Sphingomonas spp*. increased *Leptospira* growth rate [8], suggesting possible syntrophic interactions. When incubated with *Azospirillum brasilense*, viability of pathogenic *Leptospira* was enhanced at high temperature and extended under UV radiation or exposure to penicillin G, tetracycline or ampicillin. In addition, soil adsorption, thought to be an important step that favors leptospire persistence in the environment, was greatly increased in the presence of *A*. *brasilense* [8].

A major impediment to assess environmental risk for leptospirosis has been the difficulty to isolate pathogenic *Leptospira* from environmental samples, attributable in part to the fact that non-pathogenic leptospires outgrow pathogenic strains in culture. Other methods including direct animal inoculation are time-consuming, ethically questionable and have a low analytical sensitivity. However, the increasing use of molecular methods overcomes some limitations inherent to culture- and animal-based methods and provides quantitative information about the concentration of leptospires in contaminated waters [11–13].

New Caledonia provides an ideal location for studying environmental risk factors of leptospirosis because of its high leptospirosis incidence, on average 45 cases per 100,000 inhabitants, and the presence of known hot spots where annual incidence reaches up to 500 cases/100,000 population. Based on data of leptospirosis surveillance in New Caledonia, serogroup Icterohaemorrhagiae is the dominant serogroup involved in ca. 60% of human cases. Other serogroups involved in human leptospirosis include Pyrogenes (18–25%), Ballum, Australis and Pomona. Interestingly, the New Caledonian *L*. *interrogans* serovar Pyrogenes was formerly shown to be epidemiologically related to freshwater contaminations. Therefore, human leptospirosis cases infected with this strain provide opportunities to investigate the persistence and survival of pathogenic *Leptospira* in natural habitats.

The purpose of the present study was to assess the presence of pathogenic leptospires in environmental samples and to estimate their viability over time. Using a TaqMan-based real time quantitative polymerase chain reaction, we screened 73 environmental samples retrieved from 4 suspected environmental infection sites for the pathogen-specific leptospiral gene *lipL-32*. This study found that a large proportion of soil samples were positive for pathogenic leptospiral DNA, suggesting that repeated exposure to *Leptospira* may be occurring in these high-risk areas. Herein, we report findings from retrospective investigations of environmental contaminated areas to assess the presence of pathogenic *Leptospira* in order to better delineate and monitor high risk areas.

## Materials and methods

### Ethics statement

#### Patient samples, contact and authorization for interview

Institut Pasteur in New Caledonia is the country reference and only laboratory for the biological diagnosis of human leptospirosis. Biological diagnosis relies on qPCR using serum or urine as well as the reference Microagglutination Technique (MAT) using a panel of serovars of epidemiological relevance. Over the last decade, more patients have been diagnosed by qPCR, probably reflecting higher awareness and earlier medical consultation [14]. The patients were identified by a positive diagnostic qPCR targeting *lipL-32* [15]. Notification of leptospirosis to the New Caledonian Health Authority is compulsory and the infecting strain is routinely identified using a *lfb-1*-derived phylogeny of New Caledonian isolates [16] as part of the surveillance system, which also investigates cases through interviews. Only patients infected by the *L*. *interrogans* Pyrogenes were included in the study. Oral consent was obtained by the Health Authority to meet with the patient (or his parents for minors) and collect environmental samples in the suspected infection sites. Because no human sample was collected as part of the study, no written consent was required. The oral consent led to organize a site investigation. The study was approved by Institut Pasteur in New Caledonia and the ethics clearance was granted by the New Caledonian Health Authority (Direction des Affaires Sanitaires et Sociales de la Nouvelle-Calédonie).

### Study sites

**Fig 1:**
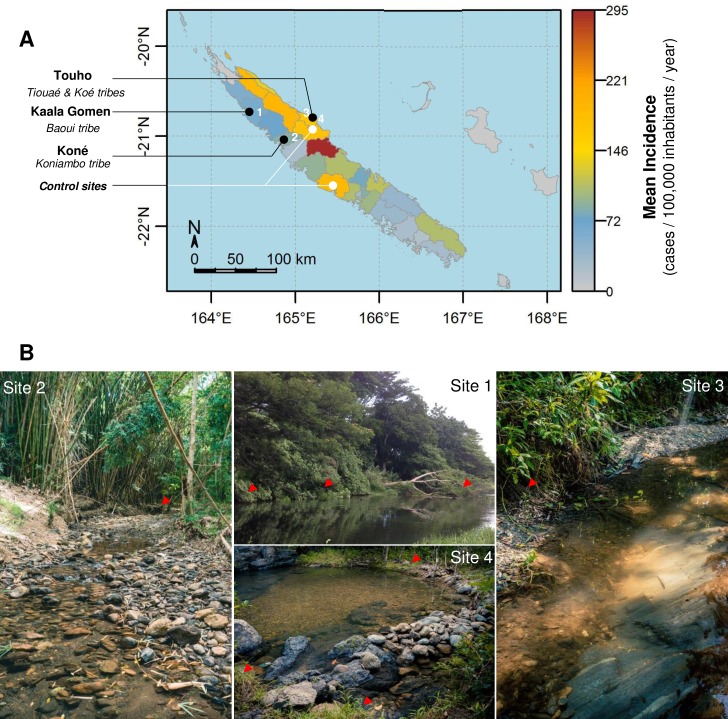
Localization of the 6 investigated environmental sites. **A.** Mapping of the 6 environmental sites investigated in this study. The map legend shows the incidence rate of leptospirosis per municipality, calculated over the 2007–2013 period. **B.** Photography showing rivers associated with activities of daily living where patients reported to be contaminated. Red arrowhead shows collected samples positive for pathogenic *Leptospira* DNA.

Four sites were identified according to the infectious strain and the good acceptance of the project by the patients and custom chiefdom (Kaala-Gomen, Koné, Touho (2 sites), Fig 1). All four study sites were within Melanesian tribes, where many outdoor activities are part of the everyday life, including fishing and bathing in freshwater streams, maintenance of backyard pig pens, hunting (deer and wild hogs). In addition, two extra sites where *L*. *interrogans* Pyrogenes was known to have been involved in former cases but where no recent contamination were reported were chosen as control sites and investigated according to the same sampling procedure. Most of the investigated sites were located in the North province of the main island where climate is sub-tropical and oceanic with a hot and rainy season from December to March (average temperature 28°C) and a cooler season from June to September (average temperature 20°C). Annual cumulative rainfall is 2400 mm on average but can range from 1460 mm to 3550 mm. Daily rainfall data for each site were obtained from the Météo France free online public database, using the nearest meteorological station for each study site.

### Collection and processing of environmental samples

Environmental investigations were started 6 to 10 weeks after the supposed infection date. Between March and June 2016, a total of 73 environmental samples were collected: 10 water samples, 52 soil samples and 11 other samples (vegetal floating debris, algae) were analyzed. Water and soil sample collections were carried out as follow: For water samples, 10 mL of subsurface water (stream or river) were collected at a 10–30 cm depth every 10 meters, alongside the water body directly into 15-mL sterile Falcon tubes, stored on ice and transported to the laboratory. For soil samples, approximately 50 g topsoil was collected from river banks (from 10 cm below to 1 meter above water level) in shaded areas using a core drilling (3 cm large by 5–7 cm height). Each soil sample was immediately placed into a 50-mL sterile Falcon tube. Water quality and environmental parameters were collected at the time of sampling (apparent meteorological and hydrological conditions, presence of iridescences, debris, foam or stagnant fludge, water color, clarity, turbidity, salinity, temperature, dissolved oxygen, pH, UV radiation, altitude). The location of sampling sites was taken with a Garmin GPS. All samples were transported to the laboratory and processed within 48 hours of collection.

### *DNA isolation* from environmental samples

Each water sample (10 mL) was centrifuged at 8000 × g for 10 min. The pellets were resuspended to a total volume of 200 μL in the original water and immediately lysed to begin the extraction process using a commercial kit (QIAamp DNA Mini Kit, Qiagen, Australia) according to the manufacturer’s instructions. DNA elution was performed with 50 μL of buffer AE. The quantity of DNA was measured by NanoDrop (Thermo Fisher Scientific). Soil samples were submitted to DNA extraction using the PowerSoil DNA Isolation kit (MO BIO), shown in preliminary experiments to be the most efficient to extract leptospiral DNA from New Caledonian soils.

Briefly, 250 mg of soil is poured in a PowerSoil bead tube before addition of 60μL suspension Buffer C1. This suspension is shaken for 5 minutes at 2,000 rpm using a MagNA Lyser (Roche). The supernatant is lysed at 4°C for 5 min with 250 μL lysis buffer C2. Up to 600 μL of supernatant is transferred in a new tube before addition of 200 μL of Inhibitory Removal Technology solution C3 before incubation at 4°C for 5 min. This step is essential for the final DNA quality as it allows the cationic flocculation of humic substances which usually account for low DNA recovery and qPCR inhibition. Up to 750 μL of the supernatant is transferred in a new tube and gently mixed with 1,200 μL of DNA binding solution C4 prior to be loaded into a Spin Filter and centrifuged at 10,000x g for 1 minute at room temperature. After washing the precipitated DNA with 500 μL of wash buffer C5 through the spin filter membrane, the DNA is eluted with 100 μL elution buffer C6.

### PCR detection of pathogenic *Leptospira spp*

Soil and water samples were tested for the presence of pathogenic *Leptospira* DNA using the real-time PCR targeting *lipL-32* [15]. The reactions were performed in a final volume of 20 μL containing 1X LightCycler 480 probes Master (Roche Applied Science, New Zealand), 0.4 μM each primer and 0.13 μM probe, and 2 μL template DNA. The cycling conditions were as described in the original publication in a LightCycler 480 (Roche Applied Science) [15].

### *Leptospira* viability qPCR assay

Samples with a positive *lipL-32* qPCR were investigated with BLU-V Viability PMA Kit (Qiagen) to evaluate the presence of viable pathogenic leptospires, except for site 1. Briefly, 5 g of soil were gently resuspended in 5 mL of 1X Phosphate Buffered Saline and let to settle down for 1 hour. Then 100 μL of this soil suspension supernatant was mixed with 2 μL of propidium monoazide (PMA; 50 μM final concentration) in light-transparent 1.5 mL microcentrifuge tubes. Following a 10 min incubation in the dark, samples were exposed for 10 min to a 3-watt LED light (460-470 nm) with gentle homogenization every 2 minutes. The sample tubes were laid horizontally under the light source to ensure optimal PMA/DNA cross-linking, thus avoiding false positive results. In order to test the efficiency of PMA treatment of membrane-compromised bacterial cells, duplicate tubes of the same soil solution supernatant were heated at 80°C for 10 min. The heat-treated samples were then cooled to room temperature before PMA addition, incubation and photoactivation. In addition, a control tube without PMA was included to determine the presence of total pathogenic *Leptospira* (both dead and live) in the soil sample. After photoinduced cross-linking, samples were treated for DNA isolation using QIAamp DNA Mini kit (Qiagen). The corresponding DNA extracts were used as templates for qPCR targeting the *lfb-1* gene [17] in order to subsequently phylogenetically identify the viable pathogenic *Leptospira* present in the sample. This qPCR was run on a LightCycler LC 2.0 using the LightCycler FastStart DNA Master SYBR Green I kit (Roche Applied Science, New Zealand) as described before [17].

### Gene sequence determination of qPCR amplicons, phylogenetic analysis and Accession Numbers

The *lfb-1* sequence polymorphism was used as a molecular phylogenetic target to link the identity of environmental leptospiral sequences to the corresponding human infecting strain. Amplified *lfb-1* DNA products obtained from environmental samples were identified by DNA sequencing. The amplicons were purified using a DNA purification kit (Qiagen, Australia) and sequenced directly as described before [16]. The resulting DNA sequence data were compared with sequences retrieved from the patient’s sample and with the GenBank database using the BLAST algorithm. The sequences obtained in this research were deposited in GenBank under the Accession Numbers: KY052025; KY052026; KY052027; KY052028; KY052029; KY052030; KY052031; KY052032; KY052033; KY052034; KY052035; KY052036; KY052037; KY052038; KY052039; KY052040.

## Results

### Pathogenic *Leptospira* are widespread in soil samples months after the index contamination event

Located in the north of New Caledonia, in places were leptospirosis is endemic, 4 different sites distributed over 3 municipalities were investigated (Fig 1). For sites 1, 2 and 4, the infections supposedly occurred on February 2^nd^, during the same heavy rain event which hit New Caledonia the same day (S1 Fig). All patients were swimming in a freshwater stream when the rain started to fall and all 3 reported an increase of the water flow and a change in water color and turbidity in their respective bathing sites. For site 3, the patient probably got infected on February 9^th^ when fishing freshwater shrimp using a mask and snorkel. For site 1, only one investigation was performed 6 weeks after the contamination event. Sites 2, 3 and 4 were investigated twice with 7 weeks (site 3 and 4) or 10 weeks (site 2) between investigations (S1 Fig).

The overall results for detection of pathogenic leptospires from these 4 sites are summarized in Table 1.

**Table 1:** Summary of sampling data and results for the six areas investigated.

	Weeks Post Infection	City	Tribe	Coordinates	Infection date	Investigation date	*lipL-32* positive samples		
							Water	Soil	Other
Site 1	6	Kaala Gomen	Baoui	20°40.823S 64°26.895E	02/02/2016	17/03/2016	**0** (4)	**6** (6)	**0** (5)
Site 2	9	Koné	Koniambo	20°59.988S	02/02/2016	06/04/2016	**0** (1)	**4** (6)	**0** (2)
	19			164°52.415E		13/06/2016	**-**	5* (7)	**-**
Control site 1	-	Bourail	Pouéo	21°30.835S 165°30.436E	**-**	06/04/2016	**0** (1)	**0** (1)	**-**
Control site 2	-	Touho	Pombei	20°54.220S 165°08.927E	**-**	11/04/2016	**0** (2)	**0** (2)	**-**
Site 3	9	North Touho	Tiouaé	20°47.572S	09/02/2016	11/04/2016	**-**	**4** (9)	**1** (2)
	16			165°08.896E		31/05/2016	**0** (2)	2* (6)	**0** (1)
Site 4	10	South Touho	Koé	20°49.090S	02/02/2016	12/04/2016	**-**	8* (9)	**1** (1)
	17			165°14.702E		31/05/2016	**-**	**1** (6)	**-**
						total	**0** (10)	**30** (52)	**2** (11)
						% positive	**0%**	**57.69%**	**12.20%**

Bold numbers represent positive samples for *lipL-32* qPCR. Numbers in brackets represent the total number of samples analyzed.

* indicates the presence of a soil sampled above water level and positive for *lipL-32* qPCR.

Interestingly, of the 10 water samples collected, none were positive for the presence of pathogenic *Leptospira* DNA by qPCR, either at the early or late investigation time point. Contrarily, soil samples were mostly positive: 58% of soil samples (30/52) were positive using *lipL-32* qPCR. It is worth to note that among soil samples investigated, we were able to amplify pathogenic *Leptospira* DNA from the river bank up to 1 meter above the water level. In such a core soil sample, DNA from pathogenic *Leptospira* was amplified from all 1-cm thick slices down to a 5-cm depth. In addition, 2 samples mostly made of benthic algae collected on the bottom of the streams were also positive using *lipL-32*-qPCR.

Despite a decreasing number of leptospiral DNA-positive soil samples in sites 3 and 4, we still successfully detected pathogenic *Leptospira* DNA in the late samples collected 4 months after the index infection event, although the qPCR Cycle Thresholds (Ct) slightly increased (S1 Fig). In contrast, in the two control sites where *L*. *interrogans* Pyrogenes was known to have been involved in human cases in previous years but without recent contamination reported (> 1 year), none of the samples collected was positive.

### Viability-PCR combined with *lfb-1* phylogenetic analysis successfully linked the presence of viable environmental leptospires to the corresponding human cases

For each soil sample positive for *lipL-32* by qPCR, we further investigated the genotype of these pathogenic leptospires using the *lfb-1* phylogenetic scheme used for patients. Viability-PCR (v-PCR) and qPCR targeting *lfb-1* using the v-PCR treated DNA as a matrix were performed subsequently when possible (for sites 2–4). The overall results for v-PCR and *lfb-1-*derived phylogenetic analysis from these 6 sites are summarized in Table 2.

**Table 2:** qPCR and v-PCR results and *lfb-1* sequence analysis for the six investigated areas.

	Weeks Post Infection	PCR *lipL-32*	PCR *lfb-1*	v-PCR	*lfb-1* Sequence

Site 1	6	**6** (15)	**5** (6)	n.d.	*L*. *interrogans* Pyrogenes
Site 2	9	**4** (9)	**2** (2)	+	*L*. *interrogans* Pyrogenes; *L*. *interrogans* Australis
	19	**5** (7)	**0** (5)	-	n.d.
Control site 1	-	**0** (2)	n.d.	n.d.	n.d.
Control site 2	-	**0** (4)	n.d.	n.d.	n.d.
Site 3	9	**5** (11)	**2** (2)	+	*L*. *interrogans* Pyrogenes
	16	**2** (9)	**2** (2)	-; -	*Leptospira spp*.
Site 4	10	**8** (10)	**3** (3)	-; +	*L*. *interrogans* Pyrogenes; *Leptospira spp*.
	17	**1** (6)	**1** (1)	-	*Leptospira spp*.

n.d.: not determined. Bold numbers represents positive samples for the corresponding qPCR. Numbers in brackets represent the total number of analyzed samples. Underlined results highlight the *lfb-1* sequences obtained using v-PCR-treated DNA as template.

**Fig 2:**
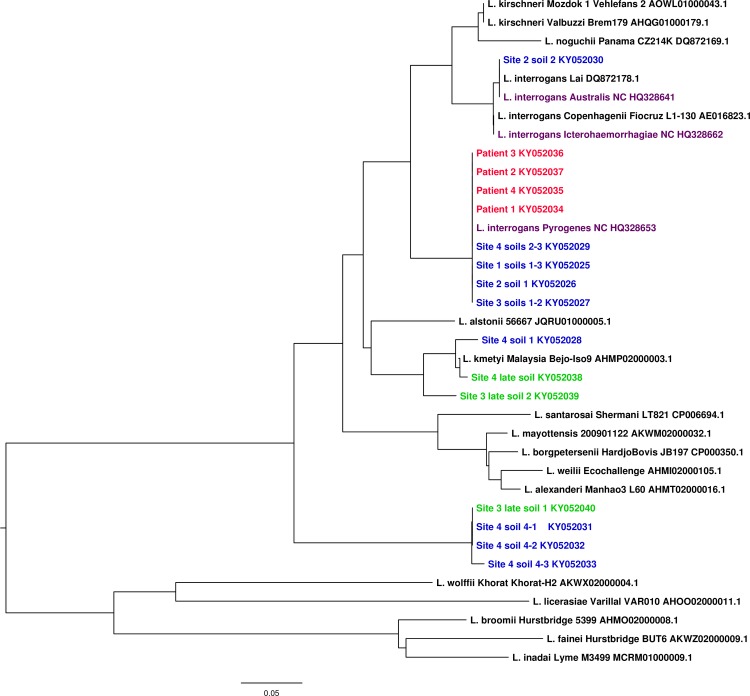
Phylogenetic analysis of leptospiral *lfb-1* gene sequences. Sequences from the 4 patients are shown in red while clones from environmental soils samples obtained during the retrospective study are shown in blue (first environmental sampling) or green (late environmental sampling). Note the presence of the *L*. *interrogans* Pyrogenes NC *lfb-1* sequence from the patient in all the corresponding investigated soils. *lfb-1* sequence from Site 2 soil 2 was identify as *L*. *interrogans* Australis and indeed cluster with *L*. *interrogans* Australis NC. Several sequences for Site 4 either cluster with *L*. *kmetyi* (Soil1) or form a new branch within pathogenic *Leptospira* species (Soil 4 sequence 1, 2 and 3). Phylogenetic tree was built using Phylo-win program with 500 bootstrap replicates applying Neighbour Joining method and Kimura’s 2-parameter distances.

In all 4 sites investigated, we were able to amplify DNA from *L*. *interrogans* Pyrogenes, respectively at 6; 9; 9 and 10 weeks post-infection (WPI) for site 1, 2, 3 and 4. To further assess if this *L*. *interrogans* Pyrogenes DNA derived from live cells, we performed a viability-PCR (for sites 2–4). Two sites (2 and 3) out of the 3 investigated were positive for v-PCR and phylogenetic analyses of the amplified DNA matched to *L*. *interrogans* Pyrogenes. Interestingly, a *lfb-1* sequence identical to a *L*. *interrogans* from serogroup Australis, involved in other human cases in New Caledonia, was also detected in site 2, concomitantly with Pyrogenes. v-PCR was also positive in site 4, but the phylogenetic analysis of the amplified *lfb-1* sequences did not match the infecting strain nor any other reported isolate (except one sequence displaying 96% identity with *L*. *kmetyi*; Fig 2).

To clarify whether the pathogenic leptospires could be detected over a longer period, soil samples were collected again 19 (site 2), 16 (site 3) or 17 WPI (site 4). All the samples investigated from these 3 sites were negative using v-PCR. However, we were still able to amplify a few *lfb-1* sequences using direct qPCR for site 3 and 4. Phylogenetic analysis of these *lfb-1* sequences appeared to differ from any known strain or species, though some were similar to those amplified during our first investigation (Fig 2).

Finally, it is interesting to note that temporal analysis of our results seems to highlight dynamic changes of the pathogenic leptospires in environmental sites. Indeed, when sequences identical to *L*. *interrogans* Pyrogenes or Australis were found during our first investigation, they were either not re-detected (site 2 and 4) or substituted by other unknown pathogenic leptospires upon our 2^nd^ investigation (site 3).

## Discussion

Infected mammals by shedding large amounts of virulent leptospires in their urine, massively contaminate their surrounding environment [5,18–20]. These pathogens eventually get drained in freshwater systems upon heavy rain episodes. This dispersion through freshwater not only participates to substantial contamination of large areas but also brings the threat right in human influence area. As environmental contamination is the major source of human leptospirosis, we attempted in this study, to evidence the presence of virulent leptospires in natural habitats in New Caledonia. Using molecular-based methods, we investigated the presence and viability of pathogenic leptospires in area believed to be contamination sites. Although the use of qPCR is becoming frequent for diagnostic purpose [14], the use of this technique on environmental samples is not commonplace, mainly because of the presence of inhibitors impairing qPCR efficiency [13].

We applied this methodology to complex environmental samples from places selected as putatively involved in human cases and we successfully amplified pathogenic *Leptospira* DNA in all the sites investigated. Phylogenetic analysis based on the *lfb-1* gene sequence successfully linked the identity of environmental leptospiral sequences to the corresponding human cases. More importantly, we demonstrated by viability qPCR that these pathogenic leptospires were viable and probably persisted in infection sites weeks after the contamination event. Notwithstanding that little is known regarding the mechanisms regulating the persistence of pathogenic leptospires in natural habitats, outside a mammalian host, general agreement in the scientific community agrees on the fact that pathogenic *Leptospira* can survive for long periods in freshwater [7] and a few studies exploring the survival of *Leptospira* in soils successfully reported re-isolation of the same *Leptospira* isolate 5 months later [21]. Our results show, that when performing retrospective investigations, pathogenic leptospires could be evidenced only in soils samples, up to 4 months after the index contamination. Though repeated contaminations from an animal source might occur, our results strongly suggest a prolonged survival in river banks and soils. Moreover, to our knowledge, this study reports the first successful viability-PCR performed on *Leptospira* from complex soil samples in combination with molecular-based typing to identify environmental leptospires involved in human cases. We formally established that up to 9 weeks after infection, the pathogenic *Leptospira* strain involved in human cases was viable in environmental soil samples, and thus potentially infectious, suggesting an ongoing risk for humans. Using samples collected 4 months after the contamination event, we were not able to evidence the presence of this particular virulent strain, therefore suggesting a decrease in *Leptospira* viability over time, a hypothesis supported by higher Ct values in qPCR from late samples (S1 Fig). These 2^nd^ investigations were performed during the cool season, also assumed to be detrimental for *Leptospira* survival. Whether this change in temperature contributed to the decrease in environmental contamination remains unknown but would be in good agreement with empirical knowledge as well as the experimental results reported by Andre-Fontaine and collaborators [7].

Interestingly, we have not evidenced pathogenic leptospires in any of our water samples, contrasting with previous observations [22,23]. Following patients’ interviews, we have investigated the suspected flowing water bodies (streams and rivers) at their normal flow rate and weeks after the index contamination event. Oppositely, stagnant water sources (gutters, wells, puddles, reservoirs) considered in other studies [22,23] were not mentioned in the interviews and therefore were not investigated. In addition, we have processed a relatively small volume of water for DNA extraction (10 mL), contrasting with larger volumes (50–1,000 mL) in other studies. Lastly, we also have investigated a smaller number of water samples compared with soils. Taken together, these facts may explain the differences observed.

The detection (or absence of detection) of live *L*. *interrogans* Pyrogenes after long periods should be interpreted with caution because of possible limitations of the v-PCR methodology, especially in environmental samples [24]. Although selective nucleic acid intercalating dyes, like propidium monoazide used in this study, represent one of the most successful recent approaches to detect viable cells (as defined by an intact cell membrane) by PCR and have been effectively evaluated in different microorganisms, major drawbacks have also been reported [24]. When applied to complex environmental samples the dye may be able to penetrate into viable or reversibly damaged cells, leading to false negative results. Further, bacteria might not all be transferred from their substratum to the supernatant or be damaged during the initial steps of the v-PCR protocol. Considering that *Leptospira* concentrations in our samples were low, v-PCR most probably underestimated bacterial viability in our experimental procedure.

Interestingly, the control sites, defined by the former presence of *L*. *interrogans* Pyrogenes but without human contamination reported over more than one year, showed no positive sample for *lipL-32* qPCR detection. This not only suggests that the index patients actually got infected in the sites investigated, but also that targeting human contamination areas is a valuable strategy to properly identify *Leptospira*-contaminated areas, notably for research purpose.

Dynamic changes in *Leptospira* population in environmental samples seem to have occurred over the time course of this study. While the infecting *L*. *interrogans* Pyrogenes could only be detected by qPCR during the first investigation, our study revealed the presence of other pathogenic *Leptospira* DNA not associated to any known species (site 4). In sites 2 and 3, *L*. *interrogans* Pyrogenes was detected alongside with other pathogenic *Leptospira spp*. It is well known that microorganisms can cooperate in complex assemblages to better exploit nutritional resources and resist to stressful environmental conditions. Because leptospires are thought to be highly susceptible to adverse environmental stresses, they could have promoted a unique microbial interaction, by which leptospires would successfully survive and persist in the environment. This emerging idea has been highlighted by the recent discovery of biofilm produced by pathogenic leptospires [25,26]. Whether multispecific biofilms either produced by *Leptospira spp*. or formed by other environmental bacteria and providing shelter to leptospires, might be present in natural habitats, contribute to the persistence and allow long-term survival of pathogenic leptospires in nutrient-poor or adverse aqueous environments deserves consideration. Recent work in other settings where leptospirosis is highly endemic supports this hypothesis [9].

Interestingly, during the flooding event which occurred on February 2^nd^, people who got infected at sites 1, 2 and 3 were bathing with at least 2 other persons who were exposed similarly to the *Leptospira* environmental risk, but did not develop a clinical form of leptospirosis. This observed low attack rate raises many questions including asymptomatic leptospirosis. A recent sero-incidence study in Brazil has shown that only a very small proportion of infections actually leads to clinical disease [27].

Overall, this study revealed that pathogenic *Leptospira* are widespread in river soils in places associated with recent human cases. The infecting strain was evidenced in all the investigated sites and viable leptospires were still detected 9 weeks after the contamination event. These observations are particularly interesting especially if they are analyzed in regards of daily rainfall data (S1 Fig). Analysis of the daily rainfall shows that in all 4 study sites, several episodes of heavy rain occurred over the 6-month period when this study was performed. But consistently with our qPCR results these rain events have probably not been a major source of re-contamination with human threatening strains, as supported by (i) the fact that a similar sampling strategy failed to evidence an environmental contamination with *L*. *interrogans* Pyrogenes in late samples and (ii) an increase in qPCR Ct values (S1 Fig), suggesting a decrease of the environmental *Leptospira* load over time. Therefore, it is likely that the *Leptospira* DNA that was detected over this 4-month study corresponded to leptospires deposited by the flooding event of February 2^nd^.

Still, temporal investigations evidenced changes in leptospiral diversity and revealed the presence of yet unreported strains in soil samples and never evidenced in any mammal in New Caledonia despite long research, suggesting that soil might act as an environmental reservoir of pathogenic leptospires offering them a protective atmosphere. v-PCR coupled to molecular-based typing on soil samples proved effective at confirming infection sites and investigating the leptospiral risk over time. Because soil DNA extraction only uses small amounts of soil, the use of this approach for risk evaluation should consider the possibility of false negative results. Still, the assessment and quantification of the leptospiral burden in environmental samples might prove valuable to guide public health interventions. To help expand the current knowledge about the leptospirosis environmental cycle and the spatial and temporal distribution of leptospires in the environment, further studies will also characterize the physicochemical characteristics of soils shown to support or oppositely compromise the survival of pathogenic leptospires. Furthermore, determination of the environmental burden may help inform health authorities before adopting preventive measures such as access restrictions to contaminated areas during heavy rainfall events. Finally, evaluation of the environmental leptospiral load through quantitative methods can be a useful method to monitor high risk areas and help protect local populations, but also to discover an unexplored biodiversity of pathogenic leptospires.

## Supporting information

S1 Fig Timeline of presumptive infection date, sampling dates and daily rainfall for the 4 sites investigated. (TIF)
