# Supplementary figures and images for: Seeking the environmental source of Leptospirosis reveals durable bacterial viability in river soils
Source: PLoS Negl Trop Dis. 2017 Feb 27;11(2):e0005414. doi: 10.1371/journal.pntd.0005414 (PMC5344526; doi:10.1371/journal.pntd.0005414)

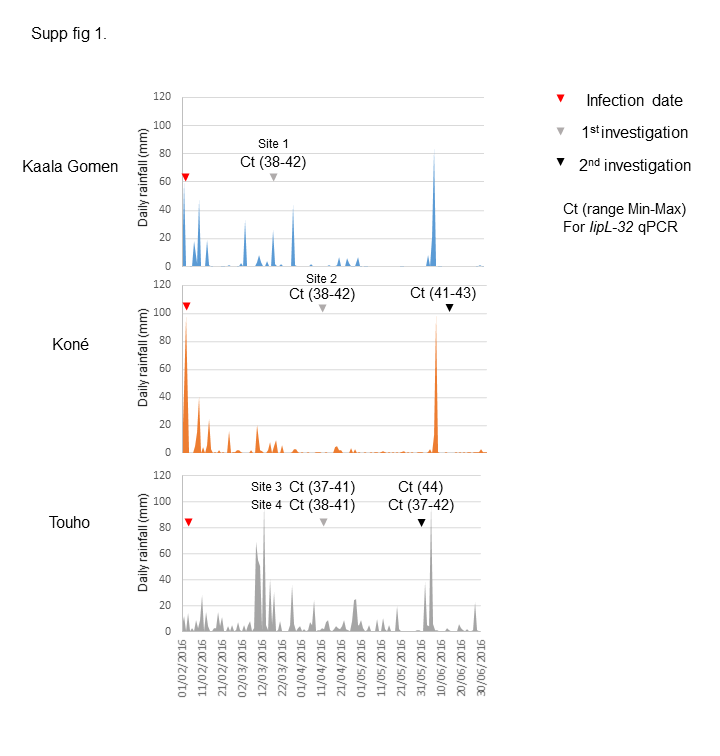

Supplement: S1 Fig — (TIF) [file pntd.0005414.s001.tif]
